# Generation and characterization of a DYNLT1-knockout mouse model reveals electrophysiological alterations and potential mechanistic contributors to atrial fibrillation

**DOI:** 10.1242/bio.061895

**Published:** 2025-06-16

**Authors:** Ting Chen, Ziyan Wang, Xinpeng You, Wenxing Guo, Yijin Chua, Qi Jiang, Yanhong Gao

**Affiliations:** ^1^ School of Food and Pharmaceutical Engineering, Nanjing Normal University, Nanjing, 210023, China; ^2^ Department of Cardiology, The Third Affiliated Hospital of Soochow University, Changzhou, 213003, China

**Keywords:** Atrial fibrillation, DYNLT1, CRISPR/Cas9, Mouse model, Atrial remodeling, TMCO1

## Abstract

Atrial fibrillation (AF) is a common arrhythmia that increases the risk of stroke and heart failure and is associated with high morbidity and mortality. However, its molecular pathogenesis remains incompletely understood. In this study, we generated a DYNLT1 knockout (KO) mouse model using CRISPR/Cas9 technology. Through electrocardiography, echocardiography, and histological analysis, we found that DYNLT1 deletion induced spontaneous AF. The KO mice exhibited not only surface electrophysiological remodeling and atrial structural changes but also increased atrial cardiomyocyte apoptosis, downregulation of gap junction proteins, and elevated inflammatory markers at the molecular level. Furthermore, using mass spectrometry, immunofluorescence, and other molecular techniques, we observed that DYNLT1 deletion reduced the distribution of its interacting protein TMCO1 in the endoplasmic reticulum (ER) of atrial cardiomyocytes, leading to ER calcium overload and potentially triggering the onset of AF. This study establishes a novel animal model for AF research, advances our understanding of the molecular mechanisms underlying AF, and provides a theoretical basis for the development of targeted molecular therapies.

## Teaser

**Summary:** This study first generated a DYNLT1-KO spontaneous atrial fibrillation (AF) mouse model via CRISPR/Cas9, highlighting the DYNLT1-TMCO1 axis in ER calcium overload and offering new insights into AF mechanisms.

## INTRODUCTION

Atrial fibrillation (AF) is the most prevalent clinical arrhythmia and is considered one of the major cardiovascular diseases of the 21st century (Linz et al., 2024). AF significantly increases the risk of stroke and heart failure, is associated with high morbidity and mortality, and imposes a substantial burden on healthcare systems (Chen et al., 2018). The pathogenesis of AF is multifactorial, involving disturbances in electrical activity, atrial structural remodeling, calcium dysregulation, and inflammatory responses (Hu et al., 2015; Nattel et al., 2020; Wijesurendra and Casadei, 2019). Genetically engineered mouse models have played a pivotal role in advancing AF research. Compared to traditional large animal models – such as goats, pigs, and dogs – which rely on rapid atrial pacing or surgical interventions to reproduce AF-like pathophysiology, their application is often limited by technical complexity and high costs (Milan and MacRae, 2005). In contrast, mouse models offer several advantages, including lower cost, higher throughput, and greater genetic tractability, thereby providing a more efficient and scalable platform for studying AF. However, spontaneous AF is rarely observed in mice, necessitating the use of genetic engineering approaches to induce stable and heritable atrial remodeling. The advent of gene-editing technologies has enabled the systemic or conditional knockout of genes such as *PITX2c* (Kirchhof et al., 2011), *Mkk4* (Davies et al., 2014), and *LKB1* (Ozcan et al., 2015), facilitating the development of murine models that recapitulate key features of AF. These findings underscore the importance of rational gene selection and the implementation of appropriate gene-editing strategies. Dynein light chain type 1 (DYNLT1), located on chromosome 6q25, encodes a light chain component of the cytoplasmic dynein complex, which mediates microtubule-based intracellular transport and participates in protein localization, signal transduction, and apoptosis (Watanabe et al., 1996; Fang et al., 2011; Hou and Witman, 2015; Tala et al., 2022; Wei et al., 2020). It is widely expressed in various tissues, including the heart (Lader et al., 1989; Xu et al., 2013), and aberrant DYNLT1 expression has been linked to diseases such as male infertility, Huntington's disease, and breast cancer (Indu et al., 2015; Rosseto et al., 2021; Huang et al., 2023). Notably, other motor protein family members like MYH7 and MYL4 have been implicated in AF pathogenesis (Orr et al., 2016; Zhang et al., 2018b), suggesting that DYNLT1 might also play a role in this disease. Based on this rationale, we hypothesized that DYNLT1 is involved in the onset or progression of AF. To test this, we generated DYNLT1 knockout mice using CRISPR/Cas9 gene-editing technology and observed spontaneous AF. We therefore explored the natural progression of AF in KO mice, including electrophysiological, structural, and functional changes, and further investigated the molecular mechanisms linking DYNLT1 deficiency to AF pathogenesis.

## RESULTS

### Generation of knockout (KO) mice using CRISPR/Cas9 technology

**Fig. 1.**
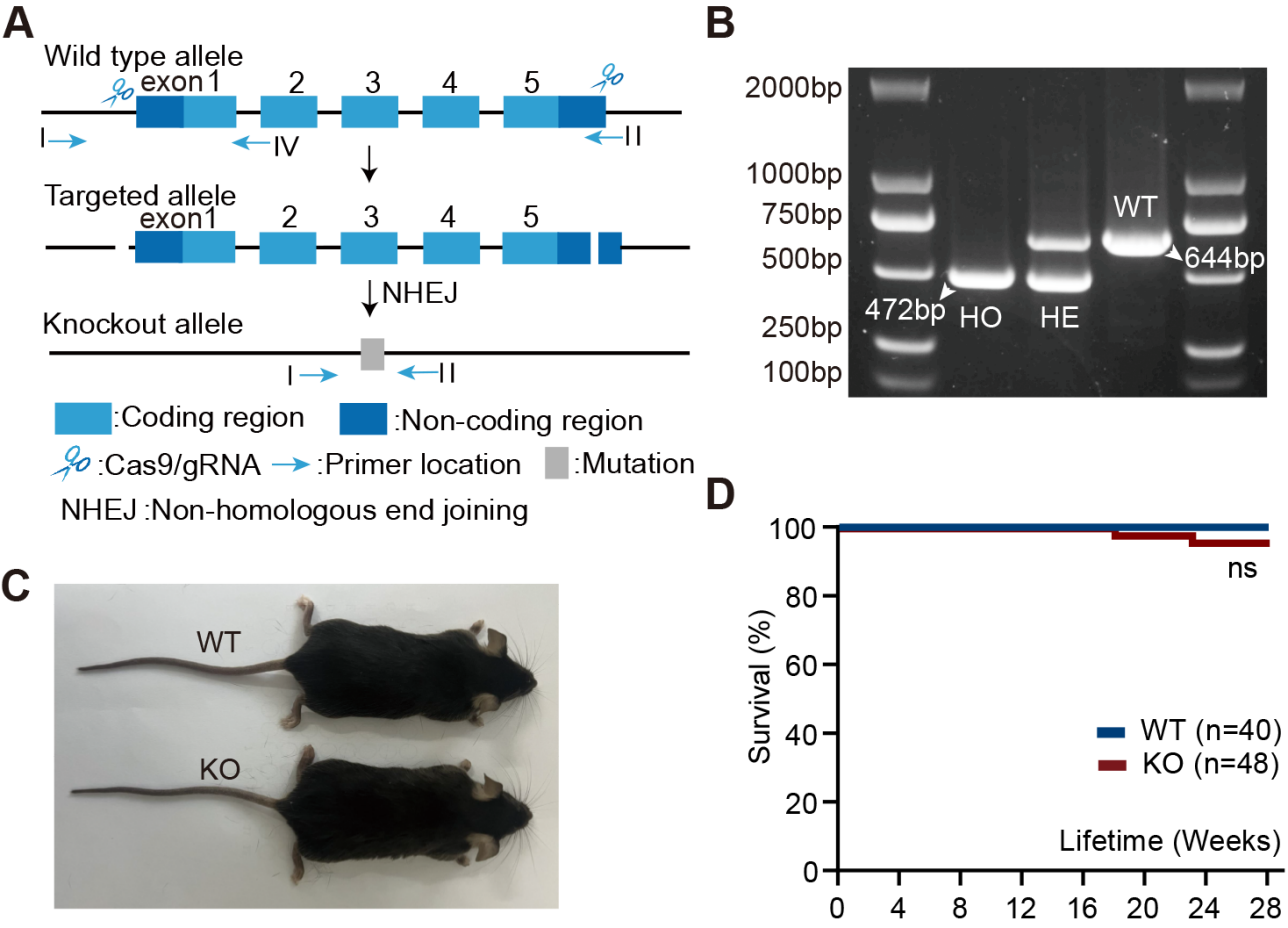
**Generation of KO mice using CRISPR/Cas9 technology.** (A) Schematic of the gene KO strategy, consisting of three main steps: (1) WT allele; (2) targeted allele; (3) KO allele. (B) The genotyping results for representative genes: WT mice (644 bp) and homozygous (HO) mice (472 bp) show different bands in PCR detection, while heterozygous (HE) mice exhibit signals at both band positions. (C) Representative gross morphology of KO and WT mice, showing no obvious differences. (D) Kaplan–Meier survival curves of WT (blue, *n*=40) and KO (red, *n*=48) mice. No statistically significant difference was observed (ns). Mice euthanized for experimental purposes were excluded from the analysis.

In this study, the strategy for constructing KO mice using CRISPR/Cas9 technology is illustrated in Fig. 1A. In the wild-type (WT) mouse genome, the target gene contains five exons (exon 1 to exon 5), with coding regions shown in light blue and non-coding regions in dark blue. To generate the knockout, Cas9/gRNA recognition sites were designed to flank exon 1 and exon 5. Following recognition and cleavage by Cas9, the DNA is repaired via the non-homologous end-joining (NHEJ) pathway, introducing deletions or disruptions in the exon regions that impair normal gene expression. This process ultimately results in a knockout allele, where the mutated regions lead to functional loss of the DYNLT1 gene. The positions of the primers used for PCR or sequencing verification are also indicated in Fig. 1A. Representative PCR results for genotype validation are shown in Fig. 1B. WT mice displayed a band at 644 bp, while homozygous KO mice showed a band at 472 bp. Heterozygous mice exhibited bands at both positions. This study focused on the differences between homozygous KO and WT mice. KO mice exhibited no apparent differences in physical appearance compared to WT controls (Fig. 1C), and there was no significant difference in survival curves between the two groups (Fig. 1D, *P*=0.1943).

### Incidence of AF and surface electrophysiological characteristics

**Fig. 2.**
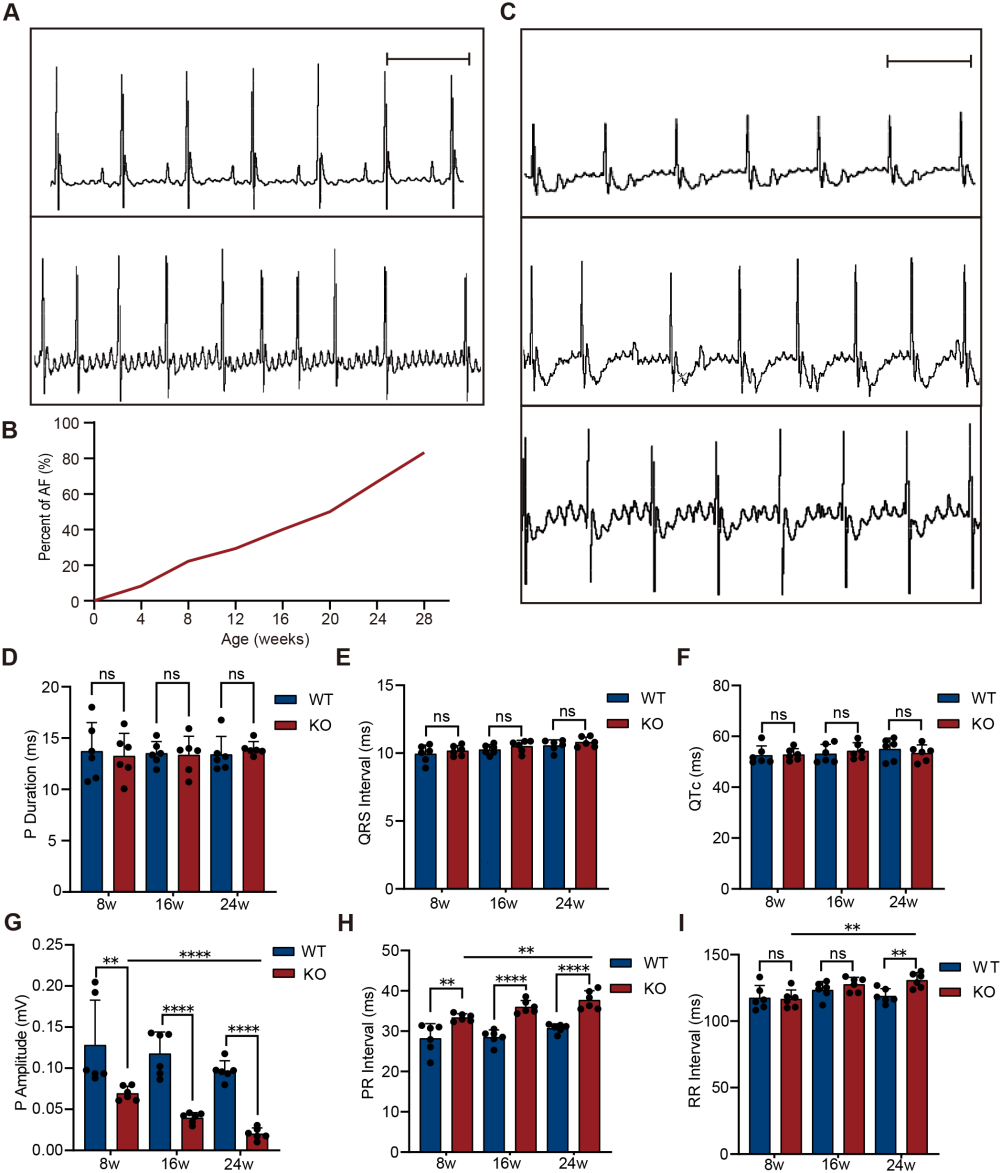
**Incidence of AF and electrocardiographic characteristics in KO mice.** (A) Representative ECG recordings. Top: normal ECG from WT mice; bottom: typical AF episode recorded in KO mice. Scale bar: 0.2 s. (B) AF incidence in KO mice increased with age, rising from 8.3% at 4 weeks to 83.3% at 28 weeks. (C) Representative ECG traces of other atrial arrhythmias in KO mice: atrioventricular block (top), atrial premature beats (middle), and atrial flutter (bottom). Scale bar: 0.2 s. (D-I) ECG analysis at 8, 16, and 24 weeks (*n*=6 per group, biological replicates). (D-F) No significant differences were observed in P-wave duration, QRS interval, or QTc between groups (two-tailed unpaired Student's *t*-test). (G) P-wave amplitude was significantly lower in KO mice compared to WT and progressively declined with age (WT 8 weeks versus KO 8 weeks, Mann–Whitney *U*-test, other comparisons were performed using Student's *t*-test). (H) PR interval (the time from the onset of the P wave to the start of the QRS complex on the ECG) was significantly prolonged in KO mice and further increased with aging (Student's *t*-test). (I) RR interval (the time between two successive R waves on the ECG) showed an age-related increase in KO mice and was significantly longer than that of WT mice at 24 weeks (Student's *t*-test). Data are presented as mean±standard deviation (s.d.). **P<0.01, ****P<0.0001; ns, not significant. All comparisons were predefined, no correction for multiple testing was applied.

ECG analysis was performed to evaluate the incidence of AF, and the surface electrophysiological characteristics of KO and WT mice aged 4 to 28 weeks. Representative ECGs are shown in Fig. 2A. The results revealed a progressive increase in AF incidence with age in KO mice: 8.3% at 4 weeks (1/12), 22.2% at 8 weeks (2/9), 29.4% at 12 weeks (5/17), 40.0% at 16 weeks (4/10), 50.0% at 20 weeks (4/8), 66.7% at 24 weeks (7/12), and 83.3% at 28 weeks (5/6) (Fig. 2B). Additionally, KO mice exhibited various types of atrial arrhythmia, including atrioventricular block (top), atrial premature beats (middle), and atrial flutter (bottom) (Fig. 2C). In contrast, WT mice showed no signs of AF or other arrhythmias.

To further assess surface ECG characteristics, ECG data from KO and WT mice at 8, 16, and 24 weeks were statistically analyzed for parameters such as P wave amplitude and duration, RR interval, PR interval, QRS duration, and corrected QT interval (QTc). The results showed no significant differences between WT and KO mice in P-wave duration, QRS interval, or QTc interval (Fig. 2D–F, *n*=6, *P*>0.05). However, compared to age-matched WT mice, the P wave amplitude in KO mice was significantly lower at 8 weeks (Fig. 2G, *n*=6, *P*=0.0022) and progressively decreased with age (8 weeks versus 24 weeks, *P*<0.0001). At 8 weeks, the PR interval in KO mice was also significantly prolonged (Fig. 2H, *n*=6, *P*=0.0069) and further worsened with age (8 weeks versus 24 weeks, *P*=0.0016). Additionally, the RR interval in KO mice showed an age-dependent increasing trend (Fig. 2I, *n*=6, 8 weeks versus 24 weeks, *P*=0.0029) and reached statistical significance at 24 weeks compared to WT mice (*P*=0.0042). These findings suggest that progressive, age-dependent electrophysiological remodeling in KO mice may be associated with the increased incidence of AF.

### Cardiac structure and function

**Fig. 3.**
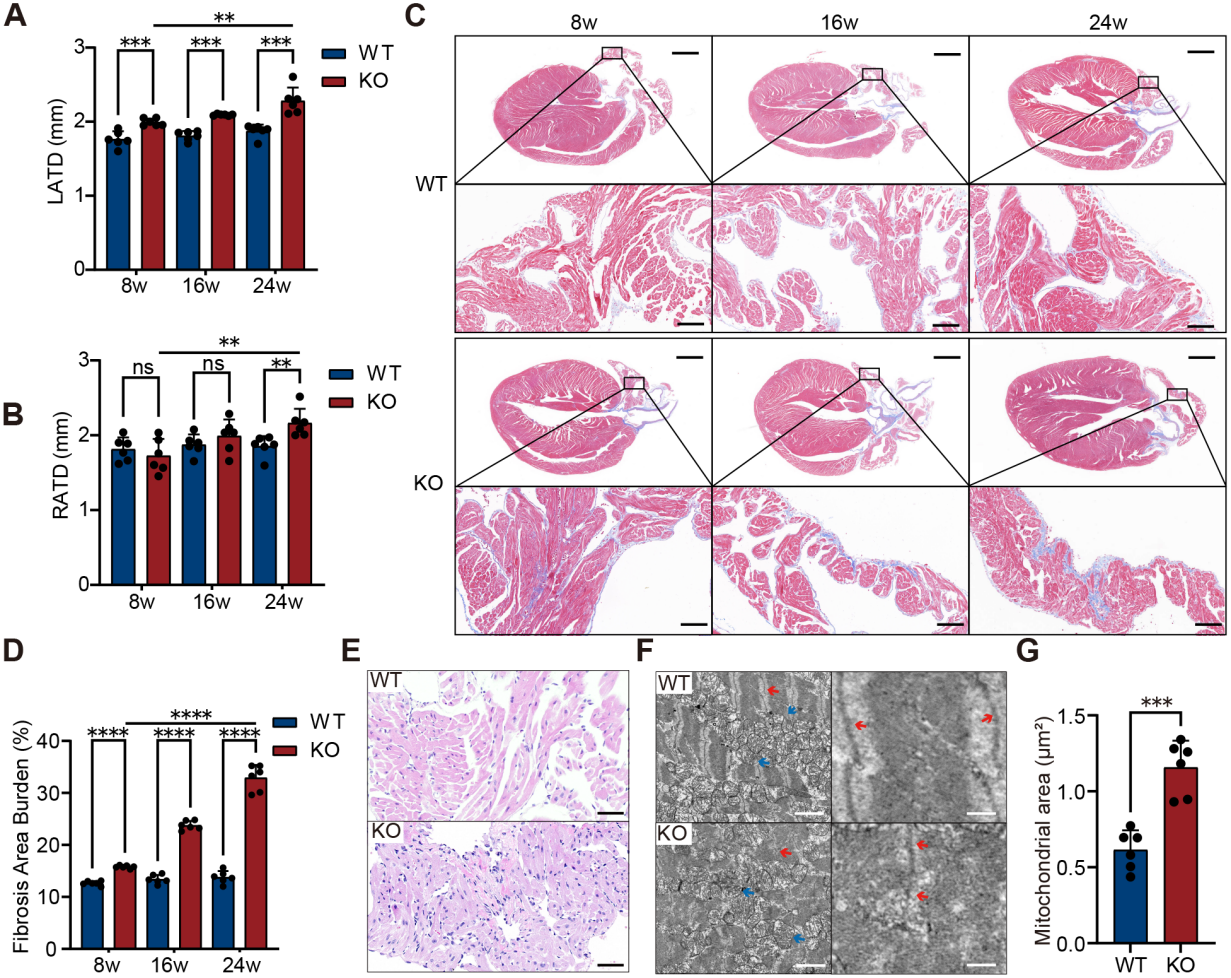
**Atrial structure and histopathological changes in KO mice.** (A) Echocardiography showed that the left atrial transverse diameter (LATD) in KO mice was significantly larger than that in WT mice at 8, 16, and 24 weeks of age, with progressive enlargement over time (*n*=6 per group; biological replicates, two-tailed unpaired Student's *t*-test). (B) The right atrial transverse diameter (RATD) increased with age in KO mice and was significantly greater than in WT mice at 24 weeks (*n*=6, Student's *t*-test). (C) Representative Masson's trichrome staining images of the left atrium from both groups of mice at 8, 16, and 24 weeks (*n*=6). Scale bar for the whole heart section: 1 mm, magnified view of the left atrium: 100 μm. (D) Quantitative analysis of left atrial fibrosis showed that the fibrosis burden in KO mice was significantly higher than that in the WT group at 8, 16, and 24 weeks, with fibrosis severity increasing with age in KO mice (*n*=6, Student's *t*-test). (E) H&E staining at 24 weeks revealed non-myocyte infiltration, disorganized cardiomyocyte arrangement, and inflammatory cell aggregation in KO mice (*n*=6). (F) TEM images of the left atrium at 24 weeks showed myofibrillar disorganization and swollen mitochondria in KO mice (blue arrows, mitochondria; red arrows, sarcomeres) (*n*=6). Scale bar: 2 µm, and the magnified image more clearly shows the disorganized sarcomeres, with a scale bar of 500 nm. (G) Quantification of average mitochondrial area in the left atrium at 24 weeks demonstrated significantly larger mitochondria in KO mice compared to WT controls (*n*=6, Student's *t*-test). Data are presented as mean±s.d. **P<0.01, ***P<0.001, ****P<0.0001; ns, not significant. No correction for multiple comparisons was applied.

**Fig. 4.**
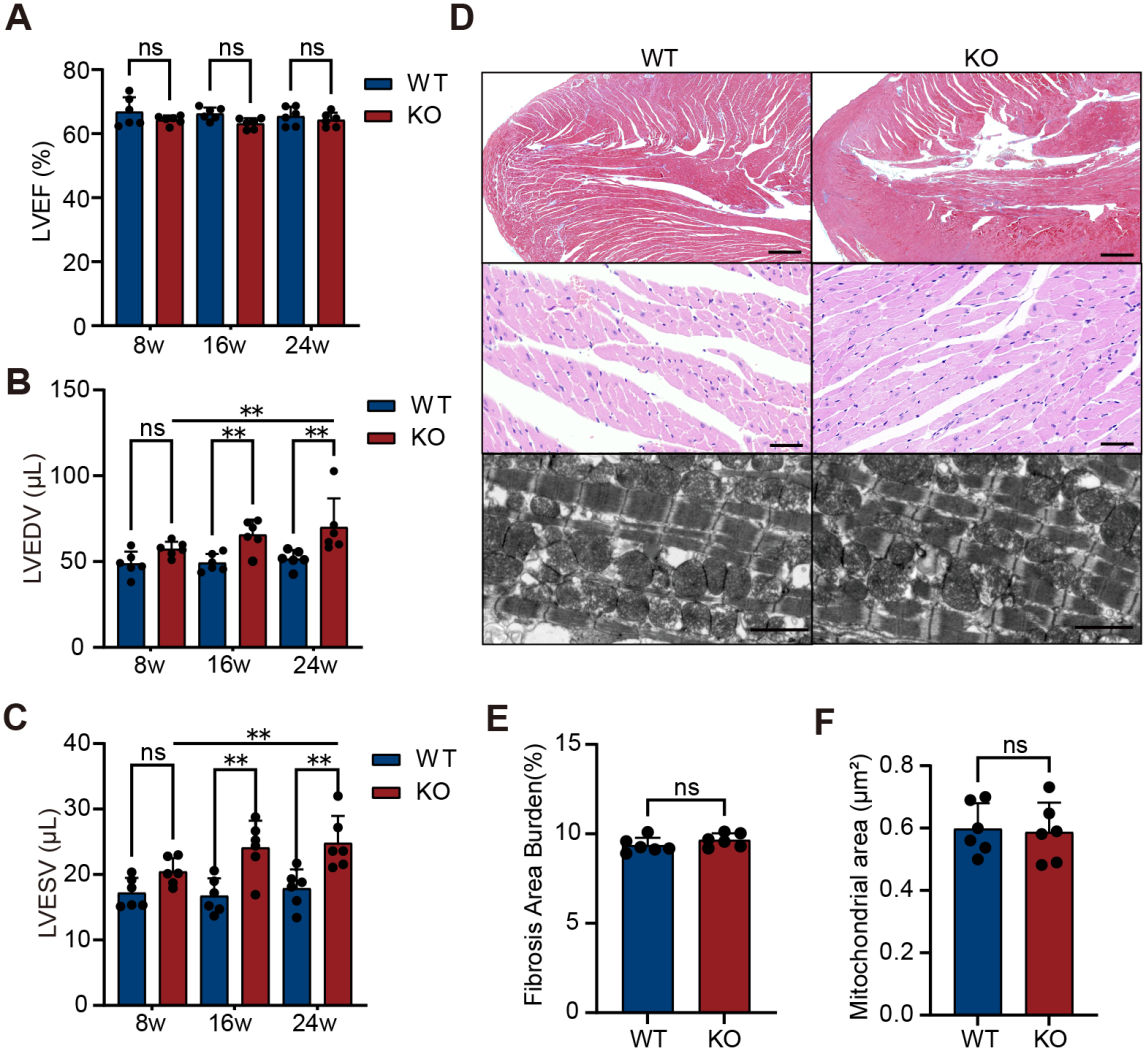
**Ventricular structure and function in KO mice.** (A) Echocardiographic analysis showed no significant difference in ejection fraction between WT and KO mice at 8, 16, and 24 weeks of age (*n*=6 per group; biological replicates, two-tailed unpaired Student's *t*-test). (B) Left ventricular end-diastolic volume (LVEDV) gradually increased with age in KO mice and was significantly higher than in WT mice at 16 weeks (*n*=6, WT 24 weeks versus KO 24 weeks, and KO 8 weeks versus KO 24 weeks, Mann–Whitney *U* test, other comparisons were performed using Student's *t*-test). (C) Left ventricular end-systolic volume (LVESV) also increased with age in KO mice, with a significant difference compared to WT mice at 16 weeks (*n*=6, Student's *t*-test). (D) Representative ventricular histology at 24 weeks (*n*=6). Top: Masson's trichrome staining revealed no significant difference in ventricular fibrosis between groups. Scale bars: 500 µm. Middle: H&E staining showed no apparent pathological abnormalities in ventricular cardiomyocytes. Scale bars: 50 µm. Bottom: TEM images showed no obvious ultrastructural changes. Scale bars: 2 μm. (E) Quantification of left ventricular fibrosis at 24 weeks showed no significant difference between KO and WT mice (*n*=6, Student's *t*-test). (F) Quantification of mitochondrial area in ventricular tissue at 24 weeks also revealed no significant difference (*n*=6, Student's *t*-test). Data are presented as mean±s.d. **P<0.01; ns, not significant. No correction for multiple comparisons was applied.

**Fig. 5.**
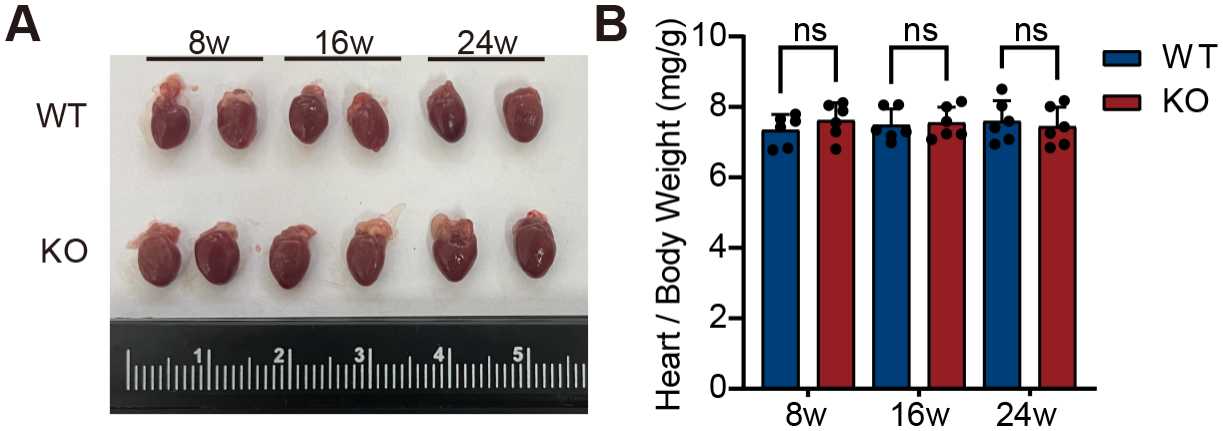
**Gross cardiac morphology in KO mice.** (A) Representative gross heart images from WT and KO mice at 8, 16, and 24 weeks of age. Each grid square on the scale equals 1 mm. (B) There was no significant difference in heart weight normalized to body weight between the two groups of mice (*n*=6 per group, biological replicates, two-tailed unpaired Student's *t*-test). Data are presented as mean±s.d. ns, not significant.

Cardiac structure and function were evaluated in KO and WT mice at 8, 16, and 24 weeks of age using echocardiography and gross anatomical assessment (*n*=6). Echocardiography results showed that, compared to age-matched WT controls, LATD was significantly larger in KO mice compared to WT mice at 8 weeks (Fig. 3A, *P*=0.0007), while RATD first reached a significant level at 24 weeks (Fig. 3B, *P*=0.0087). Additionally, both LATD and RATD in the KO group gradually increased with age (8 weeks versus 24 weeks: LATD, *P* =0.0037; RATD, *P*=0.0046). Further analysis revealed that although LVEF in the KO group remained unchanged at 24 weeks (Fig. 4A, *P*=0.4680), both LVEDV (Fig. 4B, *P*=0.0028) and LVESV (Fig. 4C, *P*=0.0040) progressively increased with age and were significantly higher than those in WT mice from 16 weeks onward. The enlargement of LVEDV and LVESV suggests that the left ventricle suggests that the left ventricle may have undergone dilative remodeling or experienced increased volume load. Gross anatomical analysis showed no significant difference in overall heart appearance or size of the hearts between the two groups of mice (Fig. 5A), and even at 24 weeks, there was no statistical difference in heart weight normalized to body weight (Fig. 5B, *P*=0.6600). Taken together, these results indicate that, compared to WT mice, KO mice exhibit significant atrial enlargement, accompanied by potential dilative remodeling or increased volume load, suggesting a gradual decline in cardiac function.

### Cardiac histopathology

In light of the structural changes and AF incidence, we further evaluated the histopathological features of the heart in KO mice (*n*=6). Masson staining revealed a marked increase in left atrial fibrosis in KO mice at 8 weeks compared to age-matched WT controls (Fig. 3C and D, *P*<0.0001), with the degree of fibrosis progressively worsening with age (*P*<0.0001). To further investigate the pathological changes in the atrial tissue of KO mice, we performed Hematoxylin and Eosin (H&E) staining and transmission electron microscopy (TEM) observations at 24 weeks. H&E staining revealed marked pathological alterations in the atrial tissue of KO mice, including infiltration of non-myocytes, myocardial disarray, and inflammatory cell aggregation (Fig. 3E, *n*=6). TEM further revealed ultrastructural abnormalities in the left atrium of KO mice, including disorganized myofibers and mitochondrial swelling (Fig. 3F and G, *P*=0.0001). These results suggest that severe damage to atrial cardiomyocytes in KO mice may play a critical role in the development of atrial dysfunction, thereby promoting the onset and progression of AF. In contrast to the marked atrial remodeling, ventricular fibrosis burden in 24-week-old KO mice showed no significant difference compared to WT controls (Fig. 4D top and 4E, *P*=0.2290). In addition, no significant pathological changes were observed in ventricular tissue under both optical microscope observation of H&E staining (Fig. 4D, middle) and TEM observation (Fig. 4D, bottom), including mitochondrial size, which showed no significant difference (Fig. 4F, *P*=0.8340). These findings suggest that pathological changes in KO mice are primarily localized to the atria, with minimal or no detectable alterations in the ventricles at this stage.

### Molecular characteristics of AF in KO mice

**Fig. 6.**
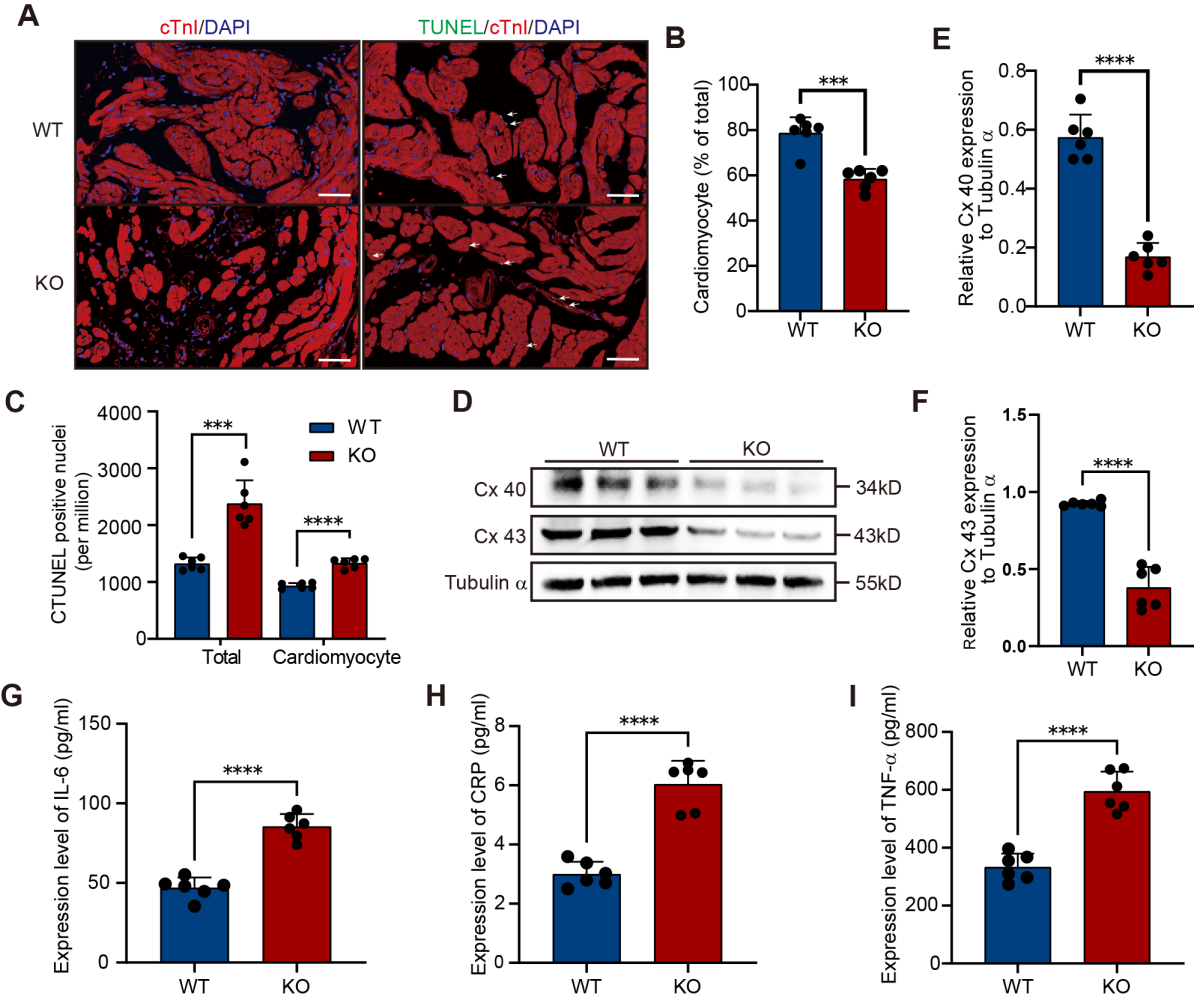
**Molecular characteristics of AF onset and progression in KO mice: enhanced apoptosis, reduced gap junction protein expression, and elevated inflammation.** (A) Representative IF images of left atrial tissue from 24-week-old WT and KO mice. Left: staining for cardiac troponin I (cTnI; red) and DAPI (blue), a fluorescent nuclear dye (4′,6-diamidino-2-phenylindole). Right: TUNEL staining highlighting apoptotic nuclei (green), indicating DNA fragmentation (terminal deoxynucleotidyl transferase dUTP nick end labeling). Arrows indicate TUNEL-positive apoptotic nuclei. Scale bars: 50 µm. (B) Quantification based on cTnI staining showed a significant reduction in the number of cardiomyocytes in KO mice (*n*=6 per group; biological replicates). (C) TUNEL staining revealed a marked increase in apoptotic nuclei and a significantly higher proportion of apoptotic cardiomyocytes in KO mice (*n*=6). (D) WB analysis of atrial tissue showing protein expression levels of Connexin 40 (Cx40, 34 kDa) and Connexin 43 (Cx43, 43 kDa), with Tubulin-α as the loading control. Full-length blots are provided in Fig. S1A. (E,F) Densitometric analysis showed that expression levels of Connexin 40 and Connexin 43 were significantly decreased in KO mice compared to WT (*n*=6). (G-I) Serum levels of interleukin-6 (IL-6), C-reactive protein (CRP), and tumor necrosis factor-α (TNF-α) were significantly elevated in KO mice compared to WT (*n*=6). All samples were collected from 24-week-old mice. Data are presented as mean±s.d. All statistical analyses were performed using two-tailed unpaired Student's *t*-tests. ***P<0.001, ****P<0.0001.

To assess the molecular alterations in the atria of KO mice, we analyzed cardiomyocyte apoptosis using combined cTnI and TUNEL staining (*n*=6). The results showed that, compared to WT mice, the number of atrial cardiomyocytes was significantly reduced in KO mice (Fig. 6A, left and 6B, *P*=0.0001), and the number of TUNEL-positive nuclei in the atrial cardiomyocytes was markedly increased (Fig. 6A, right and C, *P*<0.0001), suggesting that cardiomyocyte apoptosis may be a major contributor to cardiomyocyte loss. Furthermore, cell-to-cell coupling was severely impaired in KO mice, as indicated by significantly reduced expression of the gap junction proteins connexin 40 and connexin 43, as determined by WB analysis (Fig. 6D–F, *n*=6, *P*<0.0001). Serum inflammatory marker levels were also markedly elevated in KO mice (*n*=6), including IL-6 (Fig. 6G, *P*<0.0001), CRP (Fig. 6H, *P*<0.0001), and TNF-α (Fig. 6I, *P*<0.0001), supporting the involvement of inflammation in the atrial pathology of KO mice. In summary, the apoptosis of cardiomyocytes, reduced expression of gap junction proteins, and elevated levels of overall inflammation in the atrium of KO mice collectively constitute the important molecular characteristics of AF onset and progression.

### Molecular mechanism underlying DYNLT1 deficiency-mediated AF

**Fig. 7.**
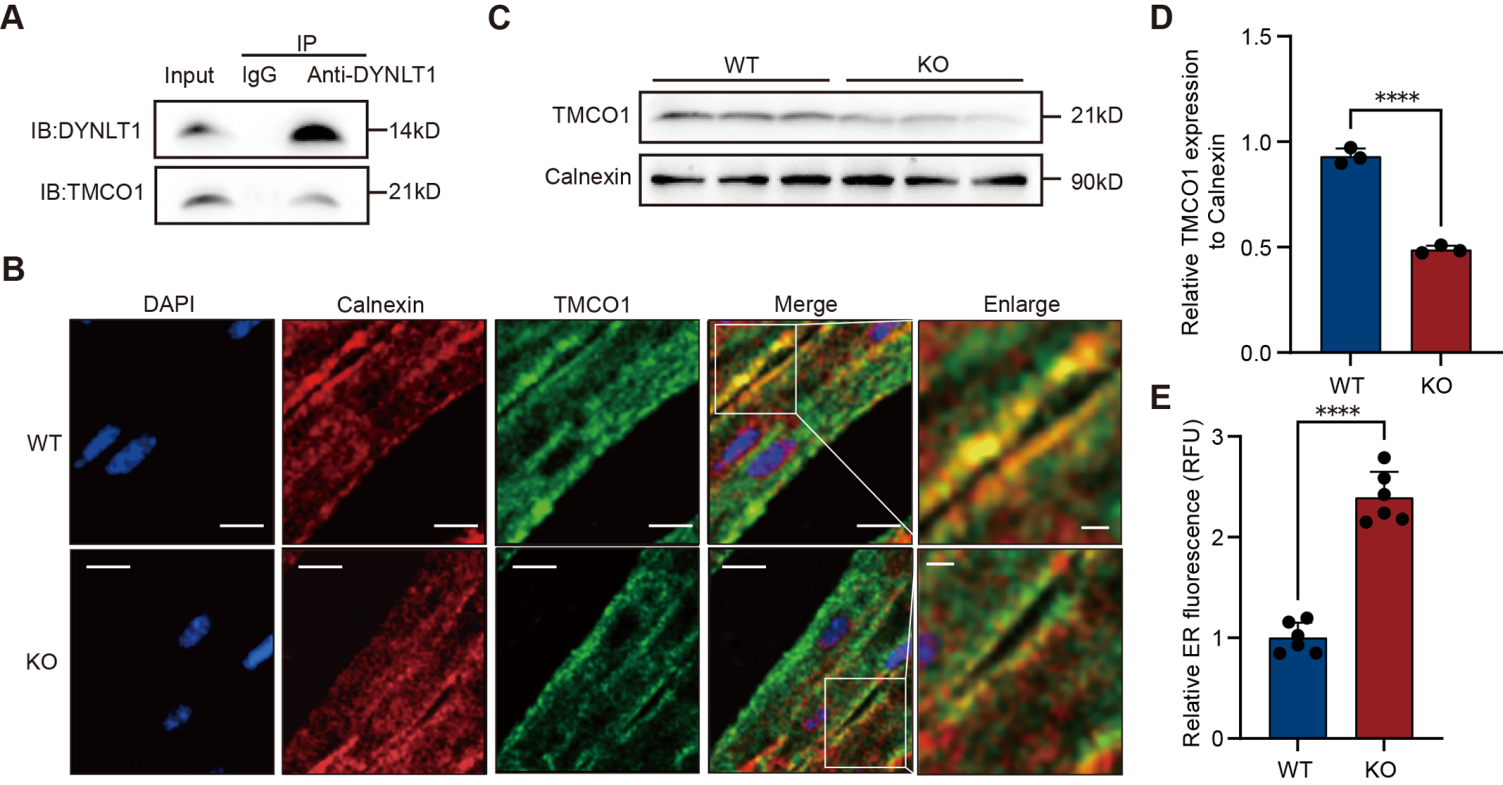
**DYNLT1 knockout may trigger AF in KO mice through TMCO1-mediated ER calcium overload in atrial myocytes.** (A) iPSC-aCMs were subjected to Co-IP using an anti-DYNLT1 antibody. Immunoblotting (IB) detected TMCO1 (21 kDa) and DYNLT1 (14 kDa) in the precipitates, but not in the IgG isotype control. The input from iPSC-aCMs confirmed normal expression of DYNLT1 and TMCO1, demonstrating their presence in the cells before the Co-IP procedure. Representative of three independent experiments. Full-length blots are provided in Fig. S1B. (B) Representative IF co-localization staining shows that in the left atrium cardiomyocytes of WT mice, TMCO1 (green) highly co-localizes with the ER marker calnexin (red) (yellow); however, in KO mice, the degree of co-localization with calnexin is reduced. DAPI (blue) was used to label the cell nuclei, *n*=6, biological replicates. Scale bars: 10 μm. The enlarged image of the merged figure shows that, compared to WT mice, the co-localization of TMCO1 with the ER is reduced in KO mice. Scale bars: 2 μm. (C) WB analysis of TMCO1 (21 kDa) in ER extracted from mouse atrial tissue calnexin (90 kDa) was used as an internal control. Each sample was prepared by pooling atrial tissues from two mice. The extracted ER fractions were characterized by minimal cytoplasmic or mitochondrial contamination. Full-length blots are provided in Fig. S1C. (D) Densitometric quantification of TMCO1, normalized to calnexin, confirmed a significant reduction in KO mice (*n*=3; ****P<0.0001, two-tailed unpaired Student's *t*-test). (E) ER calcium fluorescence intensity, measured in relative fluorescence units (RFU), was significantly higher in KO mice compared to WT, suggesting ER calcium overload (*n*=6; ****P<0.0001, Student's *t*-test). Data are presented as mean±s.d.

To further investigate the mechanism by which DYNLT1 deficiency contributes to AF, we conducted IP-MS analysis in iPSC-aCMs. TMCO1 was exclusively identified in the DYNLT1 IP group, but not in the IgG control group (Table S1), suggesting a interaction between DYNLT1 and TMCO1. Given that previous studies have largely focused on AF-related channel proteins (Feghaly et al., 2018), the presence of TMCO1 drew our attention. TMCO1 is primarily localized to the ER and acts as a calcium channel that senses excessive luminal Ca^2+^ levels and mediates its release across the ER membrane, thereby preventing calcium overload (Wang et al., 2016). To validate the interaction between DYNLT1 and TMCO1 again, we performed a Co-IP assay in iPSC-aCMs, which also confirmed an interaction between the two proteins (Fig. 7A). Considering the known role of DYNLT1 in protein trafficking, we hypothesized that DYNLT1 may facilitate the transport and anchoring of TMCO1 to the ER membrane in atrial cardiomyocytes. In the absence of DYNLT1, TMCO1 may fail to localize properly to the ER membrane, resulting in a reduction of functional TMCO1 channels and subsequent ER calcium overload. To validate this hypothesis, we examined the co-localization of TMCO1 with the endoplasmic reticulum using confocal microscopy and found that, compared to WT mice, TMCO1-ER co-localization was reduced in the atrial tissue of KO mice (Fig. 7B, *n*=6). At the same time, WB analysis showed a significant decrease in TMCO1 expression in the ER fraction of KO atrial tissue (Fig. 7C and D, *n*=3, *P*<0.0001), suggesting impaired ER localization of TMCO1 following DYNLT1 deletion. Consistently, ER calcium measurements revealed a significant increase in Ca2^+^ concentrations in the atrial cardiomyocytes of KO mice (Fig. 7E, *n*=6, *P*<0.0001), suggesting ER calcium overload. In summary, DYNLT1 deficiency leads to TMCO1-mediated ER calcium overload, which may underlie the observed cardiomyocyte apoptosis, inflammatory response, and downregulation of gap junction proteins, thereby contributing to AF onset and progression.

## DISCUSSION

In this study, we successfully generated a DYNLT1 KO mouse model using CRISPR/Cas9 technology and demonstrated that these mice spontaneously develop AF. ECG analysis revealed that surface electrical remodeling in KO mice progressively worsened with age, characterized by reduced P wave amplitude, prolonged PR and RR intervals, and increased rhythm irregularity. Further echocardiographic and histopathological examinations showed that atrial structural remodeling also progressively deteriorated with age, including enlargement of left and right atria, increased atrial fibrosis, cardiomyocyte disarray, and inflammatory cell infiltration. At the molecular level, KO mice exhibited increased cardiomyocyte apoptosis, reduced expression of the gap junction proteins connexin 40 and connexin 43, and elevated levels of inflammatory markers, including IL-6, CRP, and TNF-α. Further mechanistic explorations revealed that DYNLT1 deficiency leads to a decrease in the ER distribution of its interacting protein TMCO1 in atrial cardiomyocytes, resulting in ER calcium overload, which may trigger the onset of AF.

DYNLT1 knockout leads to reduced P wave amplitude, prolonged PR interval, and increased and irregular RR interval, indicating that DYNLT1 plays a crucial role in cardiac electrophysiological function. Previous studies have shown that reduced P wave amplitude is associated with impaired atrial depolarization or intra-atrial conduction delay, while the PR interval prolongation reflects delayed conduction through the atrioventricular node. RR interval variability may indicate sinoatrial node dysfunction or autonomic imbalance, contributing to rhythm instability (Bagliani et al., 2017; Lian et al., 2011; Nasario-Junior et al., 2018). These electrophysiological abnormalities are consistent with those observed in other AF models. For instance, LKB1-deficient mice exhibit similar changes (Ozcan et al., 2015), further supporting the notion that DYNLT1 deficiency increases susceptibility to AF through conserved mechanisms. In addition to electrophysiological changes, KO mice displayed marked atrial structural remodeling, including atrial dilation and fibrosis. Atrial enlargement may contribute to the anatomical substrate necessary for arrhythmia initiation (Marta et al., 2021), while increased fibrosis disrupts electrical conduction homogeneity, slows impulse propagation, and significantly elevates the risk of AF (Verheule and Schotten, 2021). Importantly, the pathological features observed in KO mice closely resemble those reported in human AF patients, particularly in terms of atrial dilation and fibrosis (Jansen et al., 2020). This characteristic makes the DYNLT1 KO mouse model a valuable tool for investigating the mechanisms underlying AF development.

As a key component of the dynein motor complex, DYNLT1 has not yet been reported to be associated with AF or atrial calcium overload. Although TMCO1 is recognized as a channel protein that prevents ER calcium overload, existing studies have primarily focused on developmental disorders (Alanay et al., 2014; Sun et al., 2018), and its role in ER calcium overload in atrial cardiomyocytes has not yet been reported. Our IP-MS and co-IP assays identified an interaction between DYNLT1 and TMCO1 and showed that DYNLT1 deletion impaired TMCO1 localization to the ER. This finding suggests that DYNLT1 may mediate its ER localization via dynein-mediated trafficking mechanisms. Furthermore, KO mice showed increased cardiomyocyte apoptosis, decreased expression of connexin 40 and connexin 43, and increased levels of IL-6, CRP and TNF-α. ER calcium overload is known to activate apoptotic pathways, including CHOP, caspase-12, and JNK (Lu et al., 2010). This is supported by our TUNEL staining results. Notably, uncleared apoptotic cells can release pro-inflammatory signals, contributing to secondary inflammation (Mantell et al., 1997). This aligns with our observations of inflammatory cell infiltration in the left atrium, as shown by H&E staining and elevated serum inflammatory markers. It has been reported that both apoptosis and inflammation are closely associated with structural remodeling in AF: cardiomyocyte apoptosis leads to atrial myocyte loss and fibrosis (Saraste et al., 1999), while inflammation further damages myocardial tissue and promotes fibrotic proliferation (Dobrev et al., 2023). In addition, inflammatory responses can downregulate Cx43 and Cx40, thereby disrupting intercellular electrical coupling (Willebrords et al., 2016). Thus, apoptosis, inflammation, and impaired gap junctions may be downstream effects of ER calcium overload, which synergistically contribute to the development and maintenance of AF.

Although this study has made significant progress in revealing the role of DYNLT1 in the pathogenesis of AF, there are still some limitations. First, while AF was successfully detected in KO mice, the expression of DYNLT1 has not been directly assessed in clinical samples from AF patients. This limits the clinical relevance of the findings. Future research should collect atrial appendage samples from AF patients and use techniques such as western blotting (WB) and immunohistochemistry (IHC) to detect DYNLT1 expression in these clinical samples. Second, while KO mice exhibit pathological characteristics similar to those of human AF patients, the model does not fully replicate all aspects of human AF, particularly in terms of natural pathogenesis. Future studies could consider using larger animal models (e.g. pigs or dogs) that are more clinically relevant to further validate our findings. Finally, this study focuses on the role of DYNLT1 and TMCO1 in the development of endoplasmic reticulum calcium overload in the atrium of KO mice, but the specific mechanism still requires further clarification.

## MATERIALS AND METHODS

### Materials

The mouse genotyping kit (PD101-01) was purchased from Vazyme (Nanjing, China). Anti-calnexin antibody [ab22595; 1:2000 for WB, 1:50 for immunofluorescence (IF)], Alexa Fluor 594-conjugated secondary antibody (ab150080; 1:500 for IF), and Alexa Fluor 488-conjugated secondary antibody (ab150077; 1:500 for IF) were obtained from Abcam (Cambridge, UK). The anti-DYNLT1 antibody (ab202583; 1:1000 for WB, 1:50 for IP) was also sourced from Abcam. The anti-TMCO1 antibody (AI13022; 1:500 for WB) was obtained from ABCEPTA (USA), and another anti-TMCO1 antibody for IF (NBP2-55683; 1:50) was from Novus Biologicals (USA). The anti-TOM20 antibody (A19403; 1:1000 for WB) and IgG control antibody (AC005; 1:50 for IP) were from ABclonal (Wuhan, China). anti-tubulin α antibody (10494-1-AP; 1:2000 for WB) and HRP-conjugated secondary antibody (SA00001-7H; 1:5000 for WB) were obtained from Proteintech (Chicago, IL, USA). Anti-connexin40 (sc-365107; 1:100 for WB), anti-connexin43 (sc-271837; 1:100 for WB), and anti-cTnI (sc-376662; 1:100 for IF) antibodies were from Santa Cruz Biotechnology (Dallas, TX, USA). DAPI staining reagent (G1012; 1 μg/ml), TUNEL detection kit (G1501), 4% paraformaldehyde fixative (G1101-500ML), 20× citric acid antigen retrieval solution (pH 6.0; G1202-250ML), and anti-fade mounting medium (G1401-5ML) were from Servicebio (Wuhan, China). ELISA kits for tumor necrosis factor-α (TNF-α; RX202412M), interleukin-6 (IL-6; RX203049M), and C-reactive protein (CRP; RX203387M) were obtained from RUIXIN BIOTECH (China). RIPA lysis buffer, Protein A/G magnetic beads (88802), BCA protein assay kit (23225), Coomassie Brilliant Blue R-250 stain (20278), bovine serum albumin (BSA; BP9703-100), Mag-Fluo-4-AM calcium indicator (M14206), calcium-free HBSS buffer (14175-095), acetonitrile LC-MS grade (A955-4), dithiothreitol (DTT; R0861), and formic acid LC-MS grade (28905) were from Thermo Fisher Scientific (USA). PVDF membranes (IPVH00010) were from Millipore (Burlington, MA, USA), and ECL detection reagent (FD8030) was from FDbio (Hangzhou, China). IP lysis buffer (P70100) was from NCM Biotech (China). For mass spectrometry (MS), ammonium bicarbonate (40867-50G) was from Honeywell (USA), iodoacetamide (IAA; S9827) was from Selleck (Houston, TX, USA), and MS-grade trypsin (V5280) was from Promega (USA). The endoplasmic reticulum (ER) protein extraction kit (HR8008) was purchased from Bio-Rad (Beijing, China).

### Construction and genotyping of KO Mice

In mice, the DYNLT1 homolog Dynlt1 is located on chromosome 17 and encodes a protein that shares high sequence homology with the human DYNLT1 gene (Roux et al., 1994). The KO mice were generated using the CRISPR/Cas9 system as follows: Cas9 mRNA and guide RNAs (gRNAs) targeting the DYNLT1 gene were synthesized *in vitro*. The specific gRNA sequences were as follows: gRNA1: 5′-GCGGGGCCCTAGAGCGTGGGTGG-3′, and gRNA2: 5′-GTGGCCTATTCCCGTCCTGGAGG-3′. Subsequently, a mixture of Cas9 mRNA and gRNAs was microinjected into C57BL/6J mouse zygotes, which were then implanted into pseudopregnant female mice. Approximately 20 days later, F0 mice were born. Due to the rapid development of zygotes during early division stages, the resulting F0 mice may be chimeric and may not exhibit stable inheritance. To obtain stably inherited F1 KO mice, the F0 mice, which tested positive for the knockout by PCR and sequencing, were bred with WT C57BL/6J mice. This cross produced F1 mice, which were genotyped to confirm the DYNLT1 gene knockout. The F1 mice used in this study were provided by the Shanghai Model Organisms Center, Inc, China. F1 mice were further bred to establish homozygous KO mice, which were used for all experiments. Age- and sex-matched WT mice served as controls. To determine the genotype of the mice, toe or tail tissue samples were collected at 1 week of age. Genotyping was performed according to the instructions provided with the mouse genotyping kit. DNA amplification was carried out using the 2× Taq Plus Master Mix (Dye Plus) supplied in the kit. The primer sequences for amplifying the KO and WT alleles were as follows: PⅠ (forward primer): 5′-CCACACTCAGGAGGAAACCC-3′, PⅡ (reverse primer): 5′-CTTAGGTGGCCCACAAGGAG-3′, and PⅣ (reverse primer): 5′-TGCAAATGTCTTACTCTTCCCTCA-3′. The amplification products were separated by electrophoresis on a 1% agarose gel. After electrophoresis, a UV gel imaging system (Bio-Rad GelDoc XR+) was used to visualize the agarose gel and determine the distribution of the DNA bands and genotypes.

All mice were housed under standard laboratory conditions with a controlled 12-h light/dark cycle, at a temperature of 22±2°C and humidity of 50-70%. The mice had *ad libitum* access to sterile water and standard rodent chow. Prior to the experiment, the animals were allowed to acclimate to the laboratory environment for at least 7 days. The cages were equipped with bedding to provide comfort and environmental enrichment. All experiments were conducted using KO mice and age- and sex-matched WT mice as controls. This animal study was approved by the Animal Ethics Committee of Nanjing Normal University (IACUC-2024908) and strictly adhered to the Guide for the Care and Use of Laboratory Animals (National Institutes of Health publication no. 5377-3, 1996).

### Electrocardiographic and echocardiographic assessment

Electrocardiogram (ECG) recordings were performed as previously described (Chen et al., 2024). Mice were anesthetized with isoflurane (2.5%-4.5%), placed in a supine position, and their limbs were fixed on the operating table to ensure noise-free ECG signals. Body temperature was maintained using a heating lamp. Lead II ECG recordings were obtained by placing electrodes on the right forelimb and left hindlimb. ECG signals were acquired using a rechargeable telemetry system (Kardiotek, KLR-2) and recorded with Wireless Acquisition software (Kardiotek, China). Prior to recording, isoflurane concentration was adjusted based on the heart rate to ensure it remained above 450 beats per minute. Each ECG recording session lasted more than 3 min. Data were analyzed using LabChart 8 software (ADInstruments), and the following parameters were evaluated: P wave amplitude, P wave duration, RR interval distribution, PR interval, QRS duration, and QT interval corrected for heart rate (QTc). ECG analysis was performed to identify AF and other arrhythmias. The criteria for AF identification included: (1) absence of P waves; (2) irregular RR intervals; (3) duration exceeding 10 s (Goodacre and Irons, 2002; Karpawich, 2015).

For echocardiography, mice were lightly anesthetized with 2.5%-4.5% isoflurane, and cardiac structure and function were assessed using a FUJIFILM VisualSonics Vevo F2 LT echocardiographic system equipped with a high-frequency mouse probe. Long-axis views were obtained to measure the left atrial transverse diameter (LATD) and right atrial transverse diameter (RATD). Short-axis views were used to acquire two-dimensional M-mode images of the left ventricle (LV), which were analyzed to evaluate left ventricular ejection fraction (LVEF), left ventricular end-diastolic volume (LVEDV), and left ventricular end-systolic volume (LVESV).

### Cardiac morphological and histological analysis

The animals were anesthetized with isoflurane until loss of consciousness was confirmed. This was verified by gently pinching the animals' toes, and the absence of a reflexive response confirmed unconsciousness. Once unconscious, euthanasia was performed by dislocation of the neck following anesthetization with isoflurane to ensure a rapid and humane death. After euthanasia, a gross anatomical examination was performed. The thoracic cavity was opened, and the heart was carefully excised by cutting along the root of the aorta. The heart was rinsed in pre-chilled phosphate-buffered saline (PBS) to remove surface blood, and excess adipose tissue, pericardium, and major vessels were trimmed. The overall appearance and size of the heart were documented by photographing it against a white background. The wet weight of the heart was measured using an analytical balance (Sartorius CPA225D, Sartorius) with a precision of 0.01 mg. Body weight was recorded using an electronic scale (OHAUS PA2102, OHAUS Corporation) prior to euthanasia. The heart-to-body weight ratio was calculated to normalize for differences in body size. The hearts were then used for histopathological analysis.

Mouse heart tissues were fixed in 4% paraformaldehyde at 4°C for 12 h, dehydrated using a standard protocol, paraffin-embedded, and sectioned at a thickness of 4 μm for H&E staining and Masson's trichrome staining. After staining, histopathological features were observed and analyzed using a light microscope (Nikon ECLIPSE E600). In addition, small tissue samples were taken from the left atrium and ventricle and fixed in 2.5% glutaraldehyde for TEM sample preparation (JEM-1400 Flash). After preparation, cardiomyocytes and their ultrastructural changes were observed by TEM.

To evaluate left atrial and ventricular fibrosis, three to five random fields per mouse were analyzed, covering different anatomical regions to ensure representative sampling. Fibrosis was quantified as the percentage of fibrotic area relative to total tissue area in each field, and the mean fibrotic area for the atria and ventricles was calculated for each mouse. For mitochondrial analysis, five random fields were collected from each mouse, and 30-50 mitochondrial area measurements were recorded from each field. The average mitochondrial area for each mouse was then calculated. Cardiac fibrosis burden and average mitochondrial area in mice were quantitatively measured and analyzed using ImageJ software.

### IF and western blot analysis

Paraffin-embedded mouse heart tissue sections were processed for IF analysis as described above. The TUNEL and cardiomyocyte co-labeling assay was performed and analyzed according to previously published protocols (Ozcan et al., 2015). The TUNEL detection kit, anti-cTnI antibody, Alexa Fluor 594-conjugated secondary antibody, and DAPI staining reagent used in this assay are all listed in the Materials section. Fluorescence signals were visualized and recorded using a fluorescence microscope (Nikon Eclipse C1), with cTnI (red), TUNEL-positive nuclei (green), and DAPI (blue) channels. ImageJ software was used for image processing and quantitative analysis. Sections were incubated overnight with anti-TMCO1 and anti-calnexin antibodies, followed by 1-h incubation with fluorophore-labeled secondary antibodies (Alexa Fluor 488 and 594) after PBS washing. After additional PBS washes and DAPI staining, slides were mounted and imaged using a Leica STELLARIS 5 confocal microscope. Five random fields per mouse were analyzed to ensure data reliability.

Total protein was extracted from mouse atrial tissue using RIPA lysis buffer, and protein concentration was determined with a BCA protein assay kit. Western blot (WB) analysis was performed following previously reported protocols (Zhou et al., 2024). The PVDF membranes, antibodies, and chemiluminescent substrates used in this study are listed in the Materials section. Protein bands were visualized using the ChemiDoc XRS+ imaging system (Bio-Rad), and band intensities were quantified with ImageJ software. The expression levels of target proteins were normalized to the corresponding internal control protein.

The ER fraction was isolated from mouse atrial tissue using an ER protein extraction kit. Due to the low ER protein yield from a single mouse atrium, tissues from two mice were pooled per sample. Each group included three biological replicates (a total of six WT and six KO mice). Bilateral atrial tissues were rapidly dissected, minced, and washed twice with PBS. ER isolation was performed according to the manufacturer's instructions, and the final pellet was collected as the ER fraction. The pellet was lysed using RIPA lysis buffer, followed by protein quantification, SDS-PAGE electrophoresis, and WB analysis. To verify the purity of the ER fraction, the WB membrane was first incubated with anti-TMCO1 antibody and developed. After stripping, the membrane was sequentially reprobed with antibodies against TOM20 and Tubulin-α. The membrane was then probed with anti-calnexin antibody, which displayed a clear ER-specific band. To ensure the comparability of band intensities, all WB membranes were developed using the same batch of ECL substrate and imaged under fixed exposure conditions. No brightness or contrast adjustments were made to the images. The relative expression of TMCO1 in the ER fractions of WT and KO mice was quantified by densitometric analysis and normalized to the intensity of the internal control protein calnexin.

### Mouse serum ELISA assay

Mouse serum samples were collected, and the levels of inflammatory cytokines TNF-α, IL-6, and CRP were measured using mouse serum ELISA kits. The results were read using a microplate reader (Rayto, RT-6100) to determine the concentrations of inflammatory cytokines.

### Co-immunoprecipitation and mass spectrometry analysis

Approximately 6×10^7^ human induced pluripotent stem cell-derived atrial cardiomyocytes (iPSC-aCMs) were obtained from COSMOS BIOTECH (Nanjing, China) and cultured according to the supplier's standard protocol. These cells were verified by the supplier to express cardiomyocyte-specific markers, to be free of mycoplasma and microbial contamination, and to be suitable for subsequent protein–protein interaction experiments. As previously reported (Zhang et al., 2018a), cells were lysed, and the supernatants were collected as total protein extracts. A small aliquot of the total protein was reserved as the input control, while the remaining samples were incubated with either an anti-DYNLT1 antibody or an equal amount of control IgG. Protein–antibody complexes were enriched using magnetic beads, and the bound proteins were subsequently eluted.

For IP followed by MS analysis, the eluted protein samples were separated by SDS-PAGE and stained with Coomassie Brilliant Blue. Protein bands were excised from the gel and subjected to in-gel digestion following standard protocols, including reduction with DTT, alkylation with IAA, and digestion with trypsin. The resulting peptides were extracted and reconstituted in 2% acetonitrile/0.1% formic acid for further analysis (Pan et al., 2025; Yang et al., 2019). Liquid chromatography–tandem mass spectrometry (LC-MS/MS) analysis was performed using a Thermo Fisher Scientific EASY-nLC 1200 system coupled to an Orbitrap Exploris 480 mass spectrometer. Data were acquired in data-dependent acquisition (DDA) mode. MS1 scans were recorded at a resolution of 60,000 over an m/z range of 350-1800, and MS2 scans at a resolution of 15,000, with a normalized collision energy (NCE) of 28%. Raw data were analyzed using Proteome Discoverer version 2.4 and searched against the UniProt Homo sapiens (9606) Swiss-Prot fasta database (release 20231220). Trypsin/P was used as the digestion enzyme, allowing up to two missed cleavages. Carbamidomethylation of cysteine was set as a fixed modification, and oxidation of methionine and N-terminal acetylation were set as variable modifications. Peptides were filtered with a minimum score threshold of 20 and a high-confidence setting. Based on comparison with the IgG control group, a list of candidate proteins specifically interacting with DYNLT1 was identified. In the Co-IP validation experiments, the eluted protein samples were analyzed by WB analysis. Membranes were sequentially incubated with anti-DYNLT1 and anti-TMCO1 primary antibodies, followed by incubation with a secondary antibody that facilitates the removal of IP light and heavy chains. Specific bands were detected to confirm the interaction between DYNLT1 and TMCO1. The experiment was performed in triplicate to ensure reproducibility.

### Measurement of endoplasmic reticulum calcium levels

As previously described (Blackwood et al., 2020), fresh atrial tissue was minced and digested in a solution containing 0.1% trypsin and 0.1% collagenase II. After filtration through a cell strainer and centrifugation, the supernatant was discarded. To improve atrial cardiomyocyte purity, the digested cells were resuspended in DMEM/F12 medium supplemented with 5% fetal bovine serum (FBS) and seeded into 24-well plates for 1 h to allow the rapid attachment of fibroblasts. The non-adherent cells were then collected and transferred to new wells for an additional 2-4 h of incubation to enrich atrial cardiomyocytes.

ER calcium levels were measured using the low-affinity calcium-sensitive fluorescent probe Mag-Fluo-4-AM, following a previously reported method (Chen et al., 2022). Cells were incubated with 2 μM Mag-Fluo-4-AM at 37°C for 40 min in the dark, followed by washing with HBSS buffer. Adherent cells were gently detached, centrifuged, and resuspended in calcium-free HBSS buffer at a final density of 1×10^6^ cells/ml. The stained cell suspensions were dispensed into black, clear-bottom 96-well fluorescence plates at 100 μl per well, with three technical replicates per group. Fluorescence intensity was measured using a multifunctional microplate reader with excitation and emission wavelengths set at 495 nm and 516 nm, respectively. Blank wells (no cells, no dye) and background wells (cells without dye) were included to correct for nonspecific signals and validate dye responsiveness. Final results were expressed as relative fluorescence units (RFU).

### Statistical analysis

All statistical analyses were performed using GraphPad Prism 9. Data are presented as mean±standard deviation (s.d.). Sample sizes were estimated based on prior studies to ensure adequate statistical power for detecting biologically meaningful effects. Normality of continuous variables was assessed using the Shapiro–Wilk test. For normally distributed data, two-group comparisons were performed using two-tailed unpaired Student's *t*-tests, and comparisons among three or more groups were analyzed using one-way ANOVA. For non-normally distributed data, the Mann–Whitney *U*-test (two groups) or Kruskal–Wallis test (three or more groups) was applied. Survival analysis was conducted using the Kaplan–Meier method. Animals euthanized for experimental reasons were excluded from survival curve estimation. Inclusion and exclusion criteria were predefined. Except for survival analysis, no animals or data points were excluded from the analyses. Animals were randomly assigned to experimental groups, and outcome assessments were performed by investigators blinded to group allocation. A two-tailed *P*-value <0.05 was considered statistically significant.

### Conclusion

In conclusion, this study, for the first time, demonstrates that DYNLT1 KO can lead to AF, and identifies TMCO1 as a novel player in ER calcium overload in AF, and establishes DYNLT1 KO mice as a potential new animal model for studying the disease. These findings provide a theoretical foundation for the development of molecularly targeted therapies for AF.

### Ethics approval and consent to participate

All animal procedures were conducted in accordance with the guidelines of the Institutional Animal Care and Use Committee (IACUC) of Nanjing Normal University and were approved under protocol number IACUC-2024908.

## Supplementary Material

10.1242/biolopen.061895_sup1 Supplementary information
