# Supplementary material for: Generation and characterization of a DYNLT1-knockout mouse model reveals electrophysiological alterations and potential mechanistic contributors to atrial fibrillation
Source: Biol Open. 2025 Jun 16;14(6):bio061895. doi: 10.1242/bio.061895 (PMC12208403; doi:10.1242/bio.061895)
Supplement: Supplementary information [file biolopen-14-061895-s1.pdf]

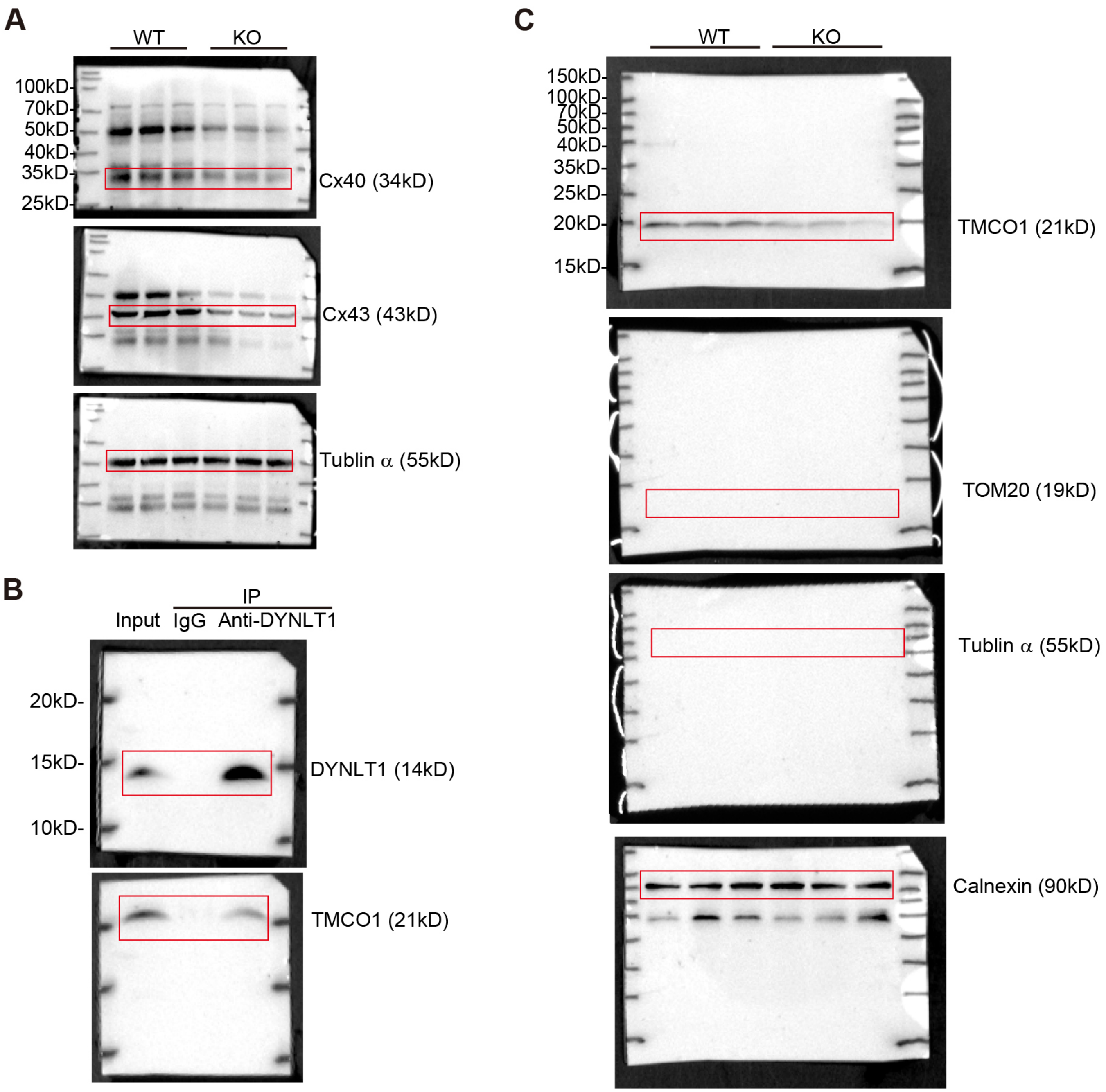
A
WT
KO
100kD-
70kD-
50kD-
40kD-
35kD-
25kD-
Cx40 (34kD)
Cx43 (43kD)
Tublin α (55kD)
B
IP
Input
IgG
Anti-DYNLT1
20kD-
15kD-
10kD-
DYNLT1 (14kD)
TMCO1 (21kD)
C
WT
KO
150kD-
100kD-
70kD-
50kD-
40kD-
35kD-
25kD-
20kD-
15kD-
TMCO1 (21kD)
TOM20 (19kD)
Tublin α (55kD)
Calnexin (90kD)

Fig. S1. Full-length, unedited Western blot images. (A) Full-length, unedited Western blot images corresponding to Fig. 6D. Blots were sequentially probed for Connexin 40 (Cx40, 34 kDa), Connexin 43 (Cx43, 43 kDa), and α-Tubulin (55 kDa), as labeled below the panels. (B) Full-length, unedited Western blot images corresponding to Fig. 7A. Blots were sequentially probed for DYNLT1 (14 kDa) and TMCO1 (21 kDa) following DYNLT1 co-immunoprecipitation. (C) Full-length, unedited Western blot images corresponding to Fig. 7C. Blots were probed sequentially with antibodies against TMCO1 (21 kDa), TOM20 (19 kDa, mitochondrial marker), α-Tubulin (55 kDa, cytoplasmic marker), and Calnexin (90 kDa, endoplasmic reticulum marker). Red boxes indicate the cropped regions displayed in the main figure. Molecular weight markers are shown on the right for reference. WT: wild-type; KO: knockout.

Table S1. DYNLT1-interacting proteins identified by mass spectrometry (MS).

Protein accession	Gene name	Protein name	MW (kDa)	Protein score	Sequence coverage (%)	Unique peptides	Group Detected
P63172	DYNLT1	Dynein light chain Tctex type 1	14	668	80	5	DYNLT1-IP only
Q9UM00	TMCO1	Calcium Load-activated calcium channel	21	44	6	1	DYNLT1-IP only

Note: Proteins were identified by MS following IP using DYNLT1 antibody. "Group Detected: DYNLT1–IP only" indicates that the protein was only detected in DYNLT1-IP samples and not in IgG control groups. MW = molecular weight; IP = immunoprecipitation.

Biology Open • Supplementary information
